# Common sampling and modeling approaches to analyzing readmission risk that ignore clustering produce misleading results

**DOI:** 10.1186/s12874-020-01162-0

**Published:** 2020-11-25

**Authors:** Huaqing Zhao, Samuel Tanner, Sherita H. Golden, Susan G. Fisher, Daniel J. Rubin

**Affiliations:** 1 Department of Clinical Sciences, Lewis Katz School of Medicine at Temple University, Philadelphia, PA 19140 USA; 2 Lewis Katz School of Medicine at Temple University, Philadelphia, PA 19140 USA; 3 Division of Endocrinology, Diabetes, and Metabolism, Welch Center for Prevention, Epidemiology, and Clinical Research, Johns Hopkins University School of Medicine, 1620 McElderry Street, Reed Hall, Room 420, Baltimore, MD 21287 USA; 4 Lewis Katz School of Medicine at Temple University, Section of Endocrinology, Diabetes, and Metabolism, 3322 N. Broad ST., Ste 205, Philadelphia, PA 19140 USA

**Keywords:** Logistic models, Patient readmission, Predictive modeling, Sampling strategies, Clustering

## Abstract

**Background:**

There is little consensus on how to sample hospitalizations and analyze multiple variables to model readmission risk. The purpose of this study was to compare readmission rates and the accuracy of predictive models based on different sampling and multivariable modeling approaches.

**Methods:**

We conducted a retrospective cohort study of 17,284 adult diabetes patients with 44,203 discharges from an urban academic medical center between 1/1/2004 and 12/31/2012. Models for all-cause 30-day readmission were developed by four strategies: logistic regression using the first discharge per patient (LR-first), logistic regression using all discharges (LR-all), generalized estimating equations (GEE) using all discharges, and cluster-weighted (CWGEE) using all discharges. Multiple sets of models were developed and internally validated across a range of sample sizes.

**Results:**

The readmission rate was 10.2% among first discharges and 20.3% among all discharges, revealing that sampling only first discharges underestimates a population’s readmission rate. Number of discharges was highly correlated with number of readmissions (r = 0.87, *P* < 0.001). Accounting for clustering with GEE and CWGEE yielded more conservative estimates of model performance than LR-all. LR-first produced falsely optimistic Brier scores. Model performance was unstable below samples of 6000–8000 discharges and stable in larger samples. GEE and CWGEE performed better in larger samples than in smaller samples.

**Conclusions:**

Hospital readmission risk models should be based on all discharges as opposed to just the first discharge per patient and utilize methods that account for clustered data.

**Supplementary Information:**

The online version contains supplementary material available at 10.1186/s12874-020-01162-0.

## Background

Recent interest in value-based care has focused attention on quality metrics in healthcare delivery. One metric, the all-cause emergency (unplanned) 30-day hospital readmission rate, has received considerable attention because it may be related to poor care, and hospitals in the US with excess readmission rates are subject to financial penalties [1, 2]. To better understand and ultimately prevent readmissions, numerous studies have examined readmission risk factors and/or developed multivariable predictive models [3–5].

Despite this growing body of literature, there is little consensus on how to sample hospitalizations and analyze multiple variables to model readmission risk. It is possible that different sampling strategies may yield different readmission rates. For example, the 30-day readmission rate for patients diagnosed with diabetes reported in the literature ranges from 10.0%, in which only the first hospitalization per patient was included as an observation, to 20.4%, in which all hospitalizations were considered as observations [6, 7]. While some variation is expected due to differences in populations across studies, sampling strategy may independently affect observed readmission rates.

In addition to variation in sampling methods, there are also multiple approaches to multivariable modeling. The most common approach is logistic regression, which treats each observation (hospital discharge) as independent [4, 5]. While this model is the least computationally demanding and the most familiar, the assumption of independence may not be valid for multiple hospitalizations of an individual patient during a given study period. The readmission risk of a patient at the time of discharge #1 is likely related to the readmission risk of the same patient at the time of discharge #2. In this case, the patient may be considered as a cluster of 2 hospital discharges. Simulation studies show that analytical approaches on clustered data that do not take into account the effects of clustering often yield erroneous results [8, 9].

One method that accounts for longitudinal correlations of data within clusters is generalized estimating equations (GEE) [10]. In the GEE approach, the intra-cluster correlation is modeled to determine the weight that should be assigned to data from each cluster [11]. If the outcome is independent of cluster size (i.e., cluster size is uninformative), then this approach is valid. However, if the outcome is related to cluster size, as is likely with readmission risk and number of discharges, then GEE may generate misleading parameter estimates. For example, people with poor dental health are likely to have fewer teeth than those with good dental health because factors that lead to poor dental health also lead to tooth loss. Therefore, in a study investigating risk factors for tooth disease, the number of teeth per person (cluster size) would be informative [11]. Cluster-weighted GEE (CWGEE) has been proposed as a valid approach to analyzing clustered data with an informative cluster size [11]. While some examples of the GEE approach exist in the readmission literature [7, 12, 13], to our knowledge, CWGEE has not been used in this context.

Herein, we compare readmission rates and predictive model accuracy of different sampling and multivariable modeling approaches in a dataset of diabetes patients previously used to develop a readmission risk prediction model [7].

## Methods

A cohort of 17,284 patients discharged from an urban academic medical center (Boston Medical Center) between 1/1/2004 and 12/31/2012 were selected, and 44,203 discharges from this cohort comprised the complete dataset. Inclusion criteria for index discharges were diabetes defined by an International Classification of Diseases, Ninth Revision, Clinical Modification (ICD-9-CM) code of 250.xx associated with hospital discharge or the presence of a diabetes-specific medication on the pre-admission medication list. Index discharges were excluded for patient age < 18 years, discharge by transfer to another hospital, discharge from an obstetric service (indicating pregnancy), inpatient death, outpatient death within 30 days of discharge, or incomplete data. Readmission documented within 8 h of a discharge was merged with the index admission to avoid counting in-hospital transfer as a readmission.

The primary outcome was all-cause readmission within 30 days of discharge. The same 46 variables previously used to develop a readmission risk prediction model were evaluated as predictors of the primary outcome to construct and validate all prediction models (see Table, Supplemental Digital Content 1, which presents patient characteristics on the variables analyzed) [7].

Five different measures of performance were assessed for each model.

1. Diagnostic discrimination (*C* statistic): the area under the receiver operating characteristic curve (AUC), for which higher values represent better discrimination [14]. Discrimination is the ability of a model to distinguish high-risk individuals from low-risk individuals [15]. The *C* statistic is the most commonly used performance measure of generalized linear regression models [16].
2. Correlation: the correlation between the observed outcome (readmission) and the value predicted by the model [17]. Unlike the *C* statistic, correlation represents a summary measure of the predictive power of a generalized linear model.
3. Coefficient of discrimination (*D*): the absolute value of the difference between model successes (the mean predicted probability of readmission, p^, for readmitted patients) and model failures (p^ for non-readmitted patients) [18]. This is a measure of overall model performance with a more intuitive interpretation for binary outcomes than the more familiar coefficient of determination (*R*^*2*^).
4. Brier score: the mean squared deviation between the predicted probability of readmission and the observed readmission rate. An overall score that captures both calibration and discrimination aspects, the Brier score can range from 0 for a perfect model to 0.25 for a noninformative model with a 50% incidence of the outcome. When the outcome incidence is lower, the maximum score for a noninformative model is lower [16, 19].
5. Scaled Brier score: the Brier score scaled by its maximum score (Brier_max_) according to the eq. 1- Brier score / Brier_max_ [16, 20]. Unlike the Brier score, the scaled Brier score is not dependent on the incidence of the outcome. For the scaled Brier score, a higher score represents greater accuracy. Brier_max_ is defined as mean(p)*(1-mean(p)) where mean(p) is the average probability of a positive outcome. The scaled Brier score is similar to Pearson’s R^2^ statistic [16]. The Brier score and scaled Brier score were chosen as measures to highlight potential differences seen when the incidence of readmission varies due to the sampling methods described below.

Sampling was performed by two methodologies. The first method included only the first index discharge per patient during the study period (first discharges). The second method included all index discharges per patient (all discharges), regardless of whether the hospitalization was a readmission relative to a prior discharge. The study sample was then divided randomly into a training sample and a validation sample [15]. The training sample, which comprised 60% of the patients in the study cohort, was used to develop the statistical prediction models. The validation sample contained the remaining 40% of the patients and was used to evaluate the performance of the prediction models.

Characteristics of the study population were described and compared between the training and validation samples. Categorical variables were presented as number (%) while continuous variables were presented as mean (standard deviation) or median (interquartile range). For the first discharge per patient dataset, the validation sample was compared to the training sample by Chi-square tests for categorical variables and two sample t-tests or Wilcoxon rank-sum tests for continuous variables. For the all discharges dataset, the validation sample was compared to the training sample by univariate generalized linear model for all variables. When analyzing all discharges, only current and prior observations available at the time of each index discharge were used for modeling.

The models can be described in mathematical terms as follows. Suppose the i^th^ patient has n_i_ observations where i = 1, 2, … N and jth discharge where j = 1, 2, …n_i_. Suppose X_ij_ is the 46-vector of covariates and Y_ij_ is the vector of discharges where Y_ij_ = 1 of i^th^ subject at j^th^ discharge readmitted within 30 days and Y_ij_ = 0 otherwise. X_ij_ can be constant over time such as gender or time-varying such as age. We further define a general class of models that specify the potential relation between readmission Y and covariates X as f (E(Y | X)) = η, where f (.) is a link function, such as the logit function, that determines the relationship between Y and X; E (Y | X) denotes the conditional mean of Y given X; and η is a function of covariates, usually a linear function such that η = *α* + *β* X where *α* and *β* are log odds ratios. We fit the readmission prevalence model assuming a logit link to estimate the effect of covariates *β* as logit(E(Y|X)) = *α* + *β* X. Then the probability of readmission within 30 days is P((Y = 1) = exp.(*α* + *β* X)/[1 + exp.(*α* + *β* X)]. This probability is estimated and used to compare performance of four statistical approaches described below.

The four approaches used to predict readmission were: 1) logistic regression using the first discharges, 2) logistic regression using all discharges, 3) GEE logistic regression with an exchangeable correlation structure using all discharges, and 4) CWGEE logistic regression with an exchangeable correlation structure using all discharges. CWGEE logistic regression is an extension of GEE logistic regression that accounts for cluster size when the outcome among observations in a cluster is dependent on the cluster size (i.e., when cluster size is informative) [11]. For each approach, univariate analyses were performed for all variables to determine those associated with 30-day readmission (*P* < 0.1). Multivariable models with best subset selection were performed to determine the adjusted associations of the variables with all-cause 30-day readmission [21, 22]. Variables associated with 30-day readmission at the *P* < 0.05 level in the multivariable models were retained.

To examine the effects of sample size on model performance, we conducted resampling studies across a range of sample sizes from 2000 to 17,000 patients by intervals of 1000. We randomly sampled each subset from the complete cohort of 17,284 patients. Each subset was then randomly divided 60% for model development and 40% for validation. For each dataset, the models were developed and compared as described above. Changes in model performance measures over sample size are displayed by line charts and compared by analysis of covariance. Lastly, we examined the relationship between the log of the number of discharges per patient (cluster size) and the number of readmissions per patient by Pearson correlation. In addition, correlations between the number of discharges per patient and predicted readmission rates were assessed. All statistical analyses were conducted using SAS 9.4 (SAS Institute, Cary, NC) and Stata 14.0 (StataCorp, College Station, TX). Institutional Review Board approval was obtained from Boston Medical Center and Temple University.

## Results

There were 17,284 patients with 44,203 discharges, of which 9034 (20.4%) were associated with 30-day readmission for any cause. The study cohort is well-distributed across middle to older adult age and sex (See Supplementary Table 1, Additional File 1). A majority of the patients are unmarried, English-speaking, insured by Medicare or Medicaid, lived within 5 miles of the hospital, educated at a high-school level or less, disabled, retired, or unemployed, and overweight or obese. This is an ethnically diverse sample, with 37.4% black, 14.7% Hispanic, and 39.7% white. The distribution of characteristics is similar between training and validation sets, with only 3 variables showing statistically significant differences despite absolute differences of < 2% (race/ethnicity [*P =* 0.03], serum sodium [*P* = 0.01], and COPD or asthma [*P* = 0.049]). Likewise, the distribution of characteristics is similar between training and validation sets of all discharges, with only 2 variables showing statistically significant differences despite absolute differences of < 2% (pre-admission sulfonylurea use [*P* = 0.032], and serum sodium [*P* = 0.029], (See Supplementary Table 2, Additional File 2).

The readmission rate is 10.2% in the sample of first discharges and 20.3% in the sample of all discharges. The AUCs are comparable among logistic regression with first discharges, logistic regression using all discharges, and GEE using all discharges, ranging from 0.803–0.821 (See Supplementary Table 3, Additional File 3, which compares performance of the four modeling methods in the whole cohort). CWGEE using all discharges yielded a lower AUC than logistic regression with first discharges and logistic regression with all discharges. Logistic regression with all discharges resulted in the highest coefficient of discrimination, followed by the GEE, first discharge and WGEE models. Analyzing all discharges both with and without GEE produced the highest predictive power by correlation, while the first discharge showed the least predictive power. Analysis of the first discharges yielded the smallest (best) Brier score while the other three methods yielded comparably higher (worse) Brier scores. Analysis of the first discharges generated the smallest (worst) scaled Brier score, which is borderline different from the largest (best) scaled Brier score produced by logistic regression with all discharges.

**Fig. 1:**
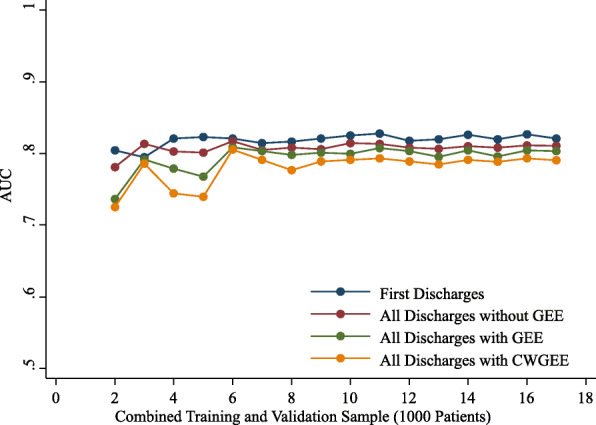
AUC of models tested in validation samples of adult patients with diabetes. The x-axis shows the size of the initial cohort from which training and validation samples were drawn. Boston, Massachusetts, 2004–2012. GEE = generalized estimating equations. CWGEE = cluster-weighted generalized estimating equations. AUC = Receiver operating characteristic area under the curve

**Fig. 2:**
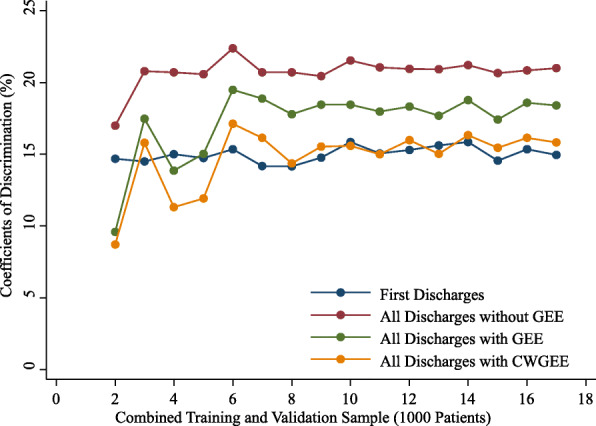
Coefficients of discrimination of models tested in validation samples of adult patients with diabetes. The x-axis shows the size of the initial cohort from which training and validation samples were drawn. Boston, Massachusetts, 2004–2012. GEE = generalized estimating equations. CWGEE = cluster-weighted generalized estimating equations

**Fig. 3:**
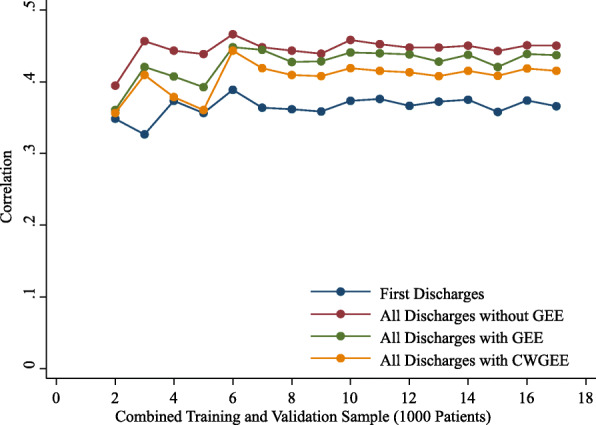
Correlation measures of models tested in validation samples of adult patients with diabetes. The x-axis shows the size of the initial cohort from which training and validation samples were drawn. Boston, Massachusetts, 2004–2012. GEE = generalized estimating equations. CWGEE = cluster-weighted generalized estimating equations

**Fig. 4:**
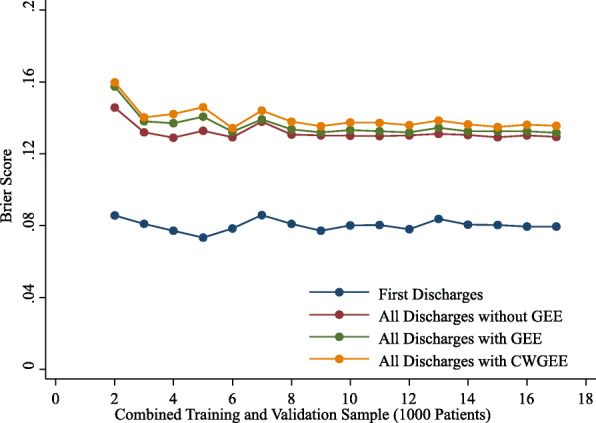
Brier scores of models tested in validation samples of adult patients with diabetes. The x-axis shows the size of the initial cohort from which training and validation samples were drawn. Boston, Massachusetts, 2004–2012. GEE = generalized estimating equations. CWGEE = cluster-weighted generalized estimating equations

**Fig. 5:**
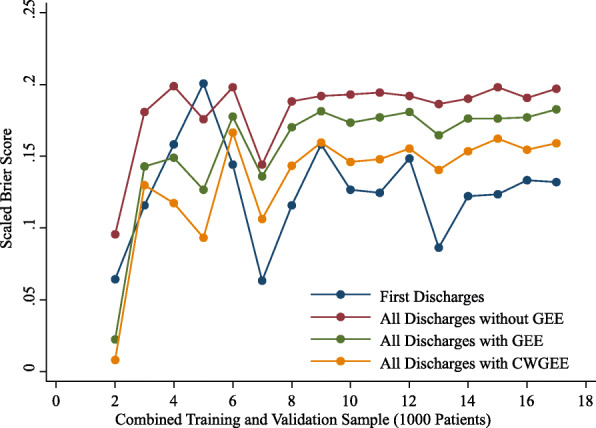
Scaled Brier scores of models tested in validation samples of adult patients with diabetes. The x-axis shows the size of the initial cohort from which training and validation samples were drawn. Boston, Massachusetts, 2004–2012. GEE = generalized estimating equations. CWGEE = cluster-weighted generalized estimating equations

The observed rates of readmission were stable with increasing sample size from 2000 to 17,000 patients for the first discharges and all discharges samples. With sample sizes less than 6000, the AUCs, coefficients of discrimination, correlations and Brier scores were variable (Figs. 1,2,3 and 4). These measures of model performance were relatively stable at sample sizes of 6000 to 17,000. The scaled Brier scores were relatively stable at sample sizes of 8000 to 17,000. The AUCs were comparable among the four approaches, with the first discharges models generally having the greatest AUC and CWGEE having the smallest AUC over the sample size range (Fig. 1). The coefficients of discrimination were greatest for the analysis of all discharges using logistic regression and generally smallest with CWGEE and first discharges (Fig. 2). The correlations were highest for logistic regression with all discharges and lowest for the first discharges (Fig. 3). The first discharges analysis yielded the lowest (best) Brier score by a substantial margin compared to the other 3 methods, which were comparable across all samples sizes (Fig. 4). In contrast, the first discharges approach generally produced the lowest (worst) scaled Brier score and all discharges logistic regression yielded the highest (best) across samples sizes (Fig. 5).

There were no statistically significant readmission rate changes over sample size for both the first discharges (*P* = 0.48) and all discharges (*P* = 0.24). The slopes for AUC, coefficients of discrimination, correlations, Brier score, and scaled Brier score were flat for both first discharges and all discharges logistic regression (See Supplementary Table 4, Additional File 4, which compares change over sample size across the model performance measures). However, the slopes for these measures were significantly different from zero for GEE and CWGEE. As sample size increases, the AUC, coefficients of discrimination, correlation measures, and scaled Brier scores increase for GEE and CWGEE, indicating more predictive accuracy. Similarly, the Brier scores decrease for GEE and CWGEE as the sample size increases, indicating greater predictive accuracy.

**Fig. 6:**
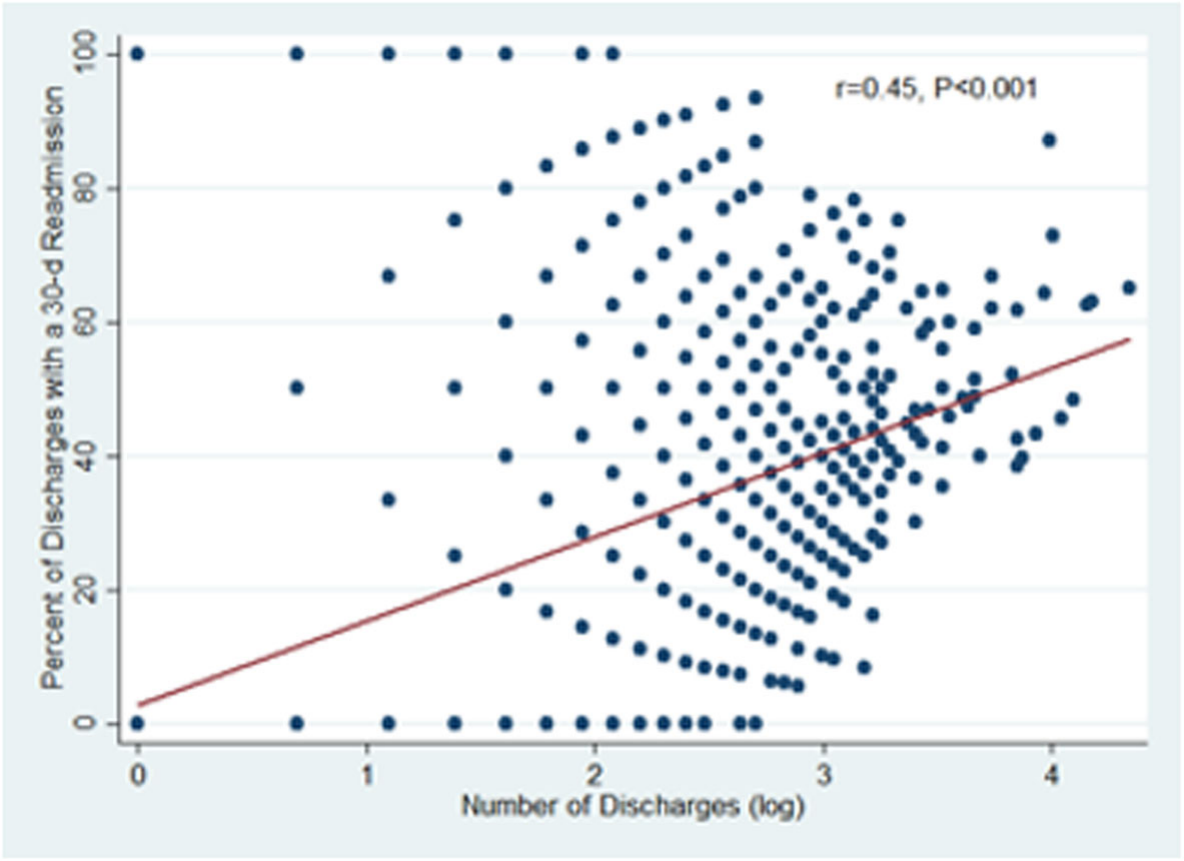
Percent of discharges followed by a readmission within 30 days by number of discharges per patient. The risk of readmission increases as the number of discharges increases (r = 0.45, *P* < 0.001 by Pearson correlation with log-transformed number of discharges)

The distribution of discharges per patient is presented in Supplementary Table 5, Additional File 5. There are 9780 (56.6%) patients with one discharge, 2936 (17.0%) with 2 discharges, and 4568 (26.4%) with at least three discharges. A total of 919 (13.3%) patients experienced one readmission and 686 (9.9%) had 2 or more readmissions. As expected, the number of discharges is positively correlated with the number of readmissions (r = 0.87, *P* < 0.001). The correlation coefficients between number of discharges and predicted readmission rates for all discharges logistic regression, GEE and CWGEE are 0.44, 0.38 and 0.31, respectively. Furthermore, the risk of readmission increases as the number of discharges increases (r = 0.45, *P* < 0.001, Fig. 6).

## Discussion

This study explored the impact of different sampling and multivariable modeling approaches on readmission risk prediction. We found that sampling only the first discharge per patient substantially underestimates the readmission rate relative to all discharges and is associated with misleading measures of model performance, particularly the Brier score. As expected, the number of readmissions per patient and the risk of readmission is highly correlated with the number of discharges per patient. In resampling studies across a range of sample sizes, most measures of model performance are unstable below a sample size of 6000 and stabilize at sample sizes of 6000 or greater. We also found that the accuracy of GEE and CWGEE is better in larger samples than in smaller samples. Lastly, we showed that using modeling approaches that account for clustering (GEE) and cluster size (CWGEE) yield generally more conservative measures of model performance than logistic regression.

Multiple methods for sampling hospitalizations and analyzing risk factors are used in studies that model predictors of readmission risk. Sampling only the first discharge per patient is a commonly used approach [6, 23–32]. This approach eliminates clusters of multiple hospitalizations per patient, enabling the valid analysis of such data by logistic regression. One disadvantage of sampling the first discharges, however, is that informative data from subsequent discharges are excluded. We show that models based on all discharges perform better on most measures of model performance than models based on first discharges. A second disadvantage of sampling only the first discharges is that the observed readmission rate is substantially lower than the readmission rate observed for all discharges during the study. In our study, the readmission rate in the sample with all discharges was nearly double the readmission rate in the first discharges sample. This occurs because excluding repeat discharges per patient minimizes the influence of patients with multiple discharges, who are at higher risk of readmission.

Such variation in observed readmission rates by different sampling methods is reflected in the literature. For example, among studies modeling readmission risk factors of patients with diabetes, we identified 14 English-language papers on PubMed published before 2019 that reported models of all-cause 30-day readmission risk [6, 7, 12, 23–28, 33–37]. Among the 7 studies that sampled all discharges in their respective datasets [7, 12, 33–37], the mean readmission rate was 18.9% with a range of 16.0–21.5%. Among the 7 studies that sampled only first discharges [6, 23–28], the mean readmission rate was 13.5% with a range of 10.0–17.1%. Given that in clinical practice a provider may be treating a patient experiencing their first hospitalization or one of many hospitalizations, we believe it is more generalizable to sample all discharges for analysis so the entire experience of the study population is captured. Although studies that sample only first discharges can provide insights into readmission risk factors, reports of readmission rates in these samples should not be broadly interpreted to represent the readmission rate for the target population. We believe this is an important methodological insight that has not been previously published.

As with approaches to sampling hospitalizations, multiple methods of modeling readmission risk are used. The most common multivariable modeling approach by far is logistic regression [4, 5]. Interestingly, logistic regression is utilized in some studies that sample all discharges without acknowledging the probable violation of the independence assumption [13, 34, 37]. In addition, some studies of readmission risk factors that utilize logistic regression do not report how hospitalizations were sampled [38–40]. The most often utilized statistical modeling approach to account for correlations within clustered hospitalization data is GEE [7, 12, 33, 41]. We are unaware of any readmission risk models that employ CWGEE.

While some studies have compared readmission risk models generated by different methods [42, 43], few have explored broader methodologic issues involved with analyzing readmissions. One study found that the LACE+ model, which was derived in a patient-level sample based on one randomly selected hospitalization per patient, performed worse in the parent sample that included all hospitalizations of the patients [44]. The authors concluded that frequent hospital utilizers probably have characteristics that were not adequately captured in the patient-level model and that capturing these characteristics may improve readmission models. Another study showed that readmission risk model performance varied by restricting samples to different reasons for readmission, that different types of data (visit history and laboratory results) contributed more predictive value than other types of data, and that limiting the cohort to patients whose index admission and readmission diagnoses matched was associated with worse model performance compared with a cohort that did not match admission and readmission diagnoses [45]. Lastly, simulation studies outside the readmission literature show that analytical approaches on clustered data that do not take into account the effects of clustering often yield erroneous results [8, 9].

Our study has some limitations. First, 30-day readmissions that may have occurred at other hospitals were not captured. However, in our cohort the 30-day readmission rate was 20.4%, which is one of the highest readmission rates reported among patients with diabetes. Therefore, it seems unlikely that a significant number of patients were readmitted elsewhere. Second, no external validation was conducted because the data were drawn from a single center. Although it is possible that the specific models generated in our study population would yield different results in other populations, the types of administrative and clinical data analyzed are likely to be generalizable to most hospitals. Therefore we believe the general concepts revealed by our findings are broadly applicable to analyses of readmissions in hospital cohorts across other settings and other chronic conditions than diabetes. Finally, we did not assess calibration by the commonly used Hosmer-Lemeshow goodness-of-fit test [46]. The standard approach with this test compares observed and predicted outcomes by decile of predicted probability. However, this test can give different results depending on the number of groups used [46]. In addition, performance of the test varies by sample size [46, 47], and 4 of the 5 measures of model performance we used incorporate calibration.

These limitations are balanced by a number of strengths. We analyzed a large enough cohort to examine performance of different modeling approaches across a broad range of sample sizes. Furthermore, this was a population well-characterized on 46 different variables used for modeling procedures. In addition, our analyses on the effects of different sampling and modeling approaches on model performance are novel in the field of readmission risk prediction.

## Conclusions

This study demonstrates the impact of different methodological approaches to analyzing hospital readmission data. All discharges in a cohort of hospitalized patients should be analyzed because sampling only the first discharge per patient produces low population estimates of readmission rates and misleading results with Brier scores. Studies should therefore transparently report how discharges are sampled. In addition, the number of readmissions per patient and the risk of readmission is highly correlated with the number of discharges per patient. Not surprisingly, modeling methods that account for clustering and cluster size yield different, generally more conservative, estimates of model performance. Researchers should be aware of the pitfalls associated with these measures of model performance and modeling procedures. Future studies of readmission risk may generate more valid results if they include all discharges and utilize modeling methods that account for clustered data.

## Supplementary Information

**Additional file 1:** **Supplementary Table 1**. Characteristics of Patients With Diabetes at the Time of the First Hospital Discharge by Training and Validation Samples, Boston, Massachusetts, 2004–2012.

**Additional file 2:** **Supplementary Table 2**. Characteristics of hospitalized patients with diabetes for all discharges by training and validation samples.

**Additional file 3:** **Supplementary Table 3**. Performance of Logistic Regression Using First Discharges (*n* = 6913), All Discharges (*n* = 17,801), All Discharges With GEE, and All Discharges With CWGEE to Predict All-Cause 30-Day Readmission Among Adults With Diabetes, Boston, Massachusetts, 2004–2012.

**Additional file 4:** **Supplementary Table 4**. Change Over Sample Size of Logistic Regression Model Performance Measures Using First Discharges (n = 6913), All Discharges (n = 17,801), All Discharges With GEE, and All Discharges With CWGEE to Predict All-Cause 30-Day Readmission in Validation Sample of Adults With Diabetes, Boston, Massachusetts, 2004–2012.

**Additional file 5:** **Supplementary Table 5**. Distribution of the Number of Discharges Per Patient (*N* = 17,801) in Sample of Adults With Diabetes, Boston, Massachusetts, 2004–2012.

## Data Availability

The data that support the findings of this study are available from Boston Medical Center but restrictions apply to the availability of these data, which were used under license for the current study, and so are not publicly available. Data are however available from the authors upon reasonable request and with permission of Boston Medical Center.

## Abbreviations

CWGEE: Cluster-weighted generalized estimating equations
GEE: Generalized estimating equations
ICD-9-CM: International Classification of Diseases, Ninth Revision, Clinical Modification
LR-first: Logistic regression using the first discharge per patient
LR-all: Logistic regression using all discharges
AUC: Area under the receiver operating characteristic curve
