# Supplementary material for: Common sampling and modeling approaches to analyzing readmission risk that ignore clustering produce misleading results
Source: BMC Med Res Methodol. 2020 Nov 25;20:281. doi: 10.1186/s12874-020-01162-0 (PMC7687737; doi:10.1186/s12874-020-01162-0)
Supplement: Supplementary file 1 — Additional file 1: Supplementary Table 1. Characteristics of Patients With Diabetes at the Time of the First Hospital Discharge by Training and Validation Samples, Boston, Massachusetts, 2004–2012. [file 12874_2020_1162_MOESM1_ESM.docx]

**Supplementary Table 1. Characteristics of Patients With Diabetes at the Time of the First Hospital Discharge by Training and Validation Samples, Boston, Massachusetts, 2004-2012.**

| Variable | All Patients  N=17284 | Training  N=10371 | Validation  N=6913 | P value |
| --- | --- | --- | --- | --- |
| Age, N (%) |  |  |  | 0.15 |
| <50 years | 3626 (21.0) | 2216 (21.4) | 1410 (20.4) |  |
| 50-59 years | 4043 (23.4) | 2377 (22.9) | 1666 (24.1) |  |
| 60-69 years | 4339 (25.1) | 2584 (24.9) | 1755 (25.4) |  |
| 70+ years | 5276 (30.5) | 3194 (30.8) | 2082 (30.1) |  |
| Gender, N (%) |  |  |  | 0.71 |
| Female | 8488 (49.1) | 5105 (49.2) | 3383 (48.9) |  |
| Male | 8796 (50.9) | 5266 (50.8) | 3530 (51.1) |  |
| Marital status, N (%) |  |  |  | 0.087 |
| Married | 6191 (35.8) | 3647 (35.2) | 2544 (36.8) |  |
| Single | 10479 (60.6) | 6349 (61.2) | 4130 (59.7) |  |
| Other or unknown | 614 (3.6) | 375 (3.6) | 239 (3.5) |  |
| Race/ethnicity, N (%) |  |  |  | 0.03 |
| Black | 6461 (37.4) | 3893 (37.5) | 2568 (37.1) |  |
| Hispanic | 2545 (14.7) | 1585 (15.3) | 960 (13.9) |  |
| White | 6863 (39.7) | 4069 (39.2) | 2794 (40.4) |  |
| Other or unknown | 1415 (8.2) | 824 (7.9) | 591 (8.5) |  |
| English speaking, N (%) |  |  |  | 0.76 |
| Yes | 13641 (78.9) | 8177 (78.8) | 5464 (79.0) |  |
| No | 3643 (21.1) | 2194 (21.2) | 1449 (21.0) |  |
| Insurance status, N (%) |  |  |  | 0.81 |
| Medicaid | 3570 (20.7) | 2122 (20.5) | 1448 (20.9) |  |
| Medicare | 7512 (43.5) | 4529 (43.7) | 2983 (43.2) |  |
| None | 1530 (8.9) | 926 (8.9) | 604 (8.7) |  |
| Private | 4672 (27.0) | 2794 (26.9) | 1878 (27.2) |  |
| Home zip code, N (%) |  |  |  | 0.38 |
| ≥5 miles from hospital | 7443 (43.1) | 4438 (42.8) | 3005 (43.5) |  |
| <5 miles from hospital | 9841 (56.9) | 5933 (57.2) | 3908 (56.5) |  |
| Education level, N (%) |  |  |  | 0.68 |
| Less than high school | 2159 (12.5) | 1295 (12.5) | 864 (12.5) |  |
| Any high school | 7735 (44.8) | 4642 (44.8) | 3093 (44.7) |  |
| Some college | 1154 (6.7) | 709 (6.8) | 445 (6.4) |  |
| College graduate | 3371 (19.5) | 1993 (19.2) | 1378 (19.9) |  |
| Unknown | 2865 (16.6) | 1732 (16.7) | 1133 (16.4) |  |
| Employment, N (%) |  |  |  | 0.49 |
| Disabled | 2167 (12.5) | 1300 (12.5) | 867 (12.5) |  |
| Employed | 2748 (15.9) | 1660 (16.0) | 1088 (15.7) |  |
| Retired | 5979 (34.6) | 3580 (34.5) | 2399 (34.7) |  |
| Unemployed | 5363 (31.0) | 3241 (31.3) | 2122 (30.7) |  |
| Other or unknown | 1027 (5.9) | 590 (5.7) | 437 (6.3) |  |
| Pre-admission sulfonylurea use, N (%) |  |  |  | 0.25 |
| Yes | 2372 (13.7) | 1398 (13.5) | 974 (14.1) |  |
| No | 14912 (86.3) | 8973 (86.5) | 5939 (85.9) |  |
| Pre-admission metformin use, N (%) |  |  |  | 0.75 |
| Yes | 5154 (29.8) | 3083 (29.7) | 2071 (30.0) |  |
| No | 12130 (70.2) | 7288 (70.3) | 4842 (70.0) |  |
| Pre-admission thiazolidinedione use, N (%) |  |  |  | 0.9 |
| Yes | 1258 (7.3) | 757 (7.3) | 501 (7.2) |  |
| No | 16026 (92.7) | 9614 (92.7) | 6412 (92.8) |  |
| Pre-admission insulin use, N (%) |  |  |  | 0.9 |
| Yes | 3516 (20.3) | 2113 (20.4) | 1403 (20.3) |  |
| No | 13768 (79.7) | 8258 (79.6) | 5510 (79.7) |  |
| Pre-admission glucocorticoid use, N (%) |  |  |  | 0.5 |
| Yes | 942 (5.5) | 575 (5.5) | 367 (5.3) |  |
| No | 16342 (94.5) | 9796 (94.5) | 6546 (94.7) |  |
| Most extreme blood glucose level, N (%) |  |  |  | 0.72 |
| 40-69 or 181-300 mg/dL | 7227 (41.8) | 4313 (41.6) | 2914 (42.2) |  |
| 70-180 mg/dL | 6848 (39.6) | 4133 (39.9) | 2715 (39.3) |  |
| <40 or >300 mg/dL | 3209 (18.6) | 1925 (18.6) | 1284 (18.6) |  |
| Diabetes inpatient consultation, N (%) |  |  |  | 0.32 |
| Yes | 2746 (15.9) | 1671 (16.1) | 1075 (15.6) |  |
| No | 14538 (84.1) | 8700 (83.9) | 5838 (84.4) |  |
| Current or prior DKA or HHS, N (%) |  |  |  | 0.63 |
| Yes | 703 (4.1) | 428 (4.1) | 275 (4.0) |  |
| No | 16581 (95.9) | 9943 (95.9) | 6638 (96.0) |  |
| Microvascular complications,^a^ N (%) |  |  |  | 0.25 |
| 0 | 15014 (86.9) | 9041 (87.2) | 5973 (86.4) |  |
| 1 | 1766 (10.2) | 1037 (10.0) | 729 (10.5) |  |
| 2 | 391 (2.3) | 221 (2.1) | 170 (2.5) |  |
| 3 | 113 (0.7) | 72 (0.7) | 41 (0.6) |  |
| Macrovascular complications,^b^ N (%) |  |  |  | 0.89 |
| 0 | 9954 (57.6) | 5978 (57.6) | 3976 (57.5) |  |
| 1 | 4988 (28.9) | 2977 (28.7) | 2011 (29.1) |  |
| 2 | 1998 (11.6) | 1210 (11.7) | 788 (11.4) |  |
| 3 | 306 (1.8) | 181 (1.7) | 125 (1.8) |  |
| 4 | 38 (0.2) | 25 (0.2) | 13 (0.2) |  |
| Pre-admission blood pressure medications, N (%) | |  |  | 0.36 |
| None | 7610 (44.0) | 4546 (43.8) | 3064 (44.3) |  |
| ACE-i or ARB | 6679 (38.6) | 4050 (39.1) | 2629 (38.0) |  |
| Non-ACE or ARB | 2995 (17.3) | 1775 (17.1) | 1220 (17.6) |  |
| Pre-admission statin use, N (%) |  |  |  | 0.47 |
| Yes | 6459 (37.4) | 3898 (37.6) | 2561 (37.0) |  |
| No | 10825 (62.6) | 6473 (62.4) | 4352 (63.0) |  |
| White blood cell count, N (%) |  |  |  | 0.084 |
| Low <4 K/μL | 552 (3.2) | 329 (3.2) | 223 (3.2) |  |
| Normal 4-11 K/μL | 13216 (76.5) | 7989 (77.0) | 5227 (75.6) |  |
| High >11 K/μL | 3516 (20.3) | 2053 (19.8) | 1463 (21.2) |  |
| Hematocrit (%), mean (SD) | 34.5 (5.29) | 34.5 (5.26) | 34.6 (5.32) | 0.21 |
| Serum albumin, N (%) |  |  |  | 0.13 |
| 4+ g/dL | 15234 (34.5) | 9024 (34.2) | 6210 (34.9) |  |
| <4 g/dL | 23982 (54.3) | 14368 (54.4) | 9614 (54.0) |  |
| Unknown | 4987 (11.3) | 3010 (11.4) | 1977 (11.1) |  |
| Serum sodium, N (%) |  |  |  | 0.01 |
| Low <135 mmol/L | 1708 (9.9) | 985 (9.5) | 723 (10.5) |  |
| Normal 135-145 mmol/L | 15412 (89.2) | 9301 (89.7) | 6111 (88.4) |  |
| High >145 mmol/L | 164 (0.9) | 85 (0.8) | 79 (1.1) |  |
| Serum potassium, N (%) |  |  |  | 0.25 |
| Low <3.1 mmol/L | 174 (1.0) | 110 (1.1) | 64 (0.9) |  |
| Normal 3.1-5.3 mmol/L | 16153 (93.5) | 9666 (93.2) | 6487 (93.8) |  |
| High >5.3 mmol/L | 957 (5.5) | 595 (5.7) | 362 (5.2) |  |
| Serum creatinine (mg/dL), median (IQR) | 0.89 (0.7, 1.12) | 0.89 (0.7-1.12) | 0.9 (0.7, 1.13) | 0.3 |
| Body mass index, N (%) |  |  |  | 0.42 |
| <18.5 kg/m^2^ | 286 (1.7) | 167 (1.6) | 119 (1.7) |  |
| 18.5 – 24.9 kg/m^2^ | 2666 (15.4) | 1595 (15.4) | 1071 (15.5) |  |
| 25.0 – 29.9 kg/m^2^ | 5073 (29.4) | 3091 (29.8) | 1982 (28.7) |  |
| ≥30.0 kg/m^2^ | 9259 (53.6) | 5518 (53.2) | 3741 (54.1) |  |
| Discharged 90 days prior to admission, N (%) | |  |  | 0.13 |
| Yes | 1179 (6.8) | 732 (7.1) | 447 (6.5) |  |
| No | 16105 (93.2) | 9639 (92.9) | 6466 (93.5) |  |
| Discharge 1 year prior to admission, N (%) |  |  |  | 0.84 |
| Home | 1554 (9.0) | 935 (9.0) | 619 (9.0) |  |
| Home with nursing care | 442 (2.6) | 272 (2.6) | 170 (2.5) |  |
| Sub-acute facility | 409 (2.4) | 236 (2.3) | 173 (2.5) |  |
| Against medical advice | 48 (0.3) | 28 (0.3) | 20 (0.3) |  |
| No discharge recorded | 14831 (85.8) | 8900 (85.8) | 5931 (85.8) |  |
| Urgent or emergent admission, N (%) |  |  |  | 0.62 |
| Yes | 13792 (79.8) | 8263 (79.7) | 5529 (80.0) |  |
| No | 3492 (20.2) | 2108 (20.3) | 1384 (20.0) |  |
| Intensive care admission, N (%) |  |  |  | 0.48 |
| Yes | 3137 (18.1) | 1900 (18.3) | 1237 (17.9) |  |
| No | 14147 (81.9) | 8471 (81.7) | 5676 (82.1) |  |
| Blood transfusion given, N (%) |  |  |  | 0.37 |
| Yes | 2279 (13.2) | 1387 (13.4) | 892 (12.9) |  |
| No | 15005 (86.8) | 8984 (86.6) | 6021 (87.1) |  |
| Parenteral or enteral nutrition, N (%) |  |  |  | 0.07 |
| Yes | 580 (3.4) | 327 (3.2) | 253 (3.7) |  |
| No | 16704 (96.6) | 10044 (96.8) | 6660 (96.3) |  |
| Depression or psychosis ever, N (%) |  |  |  | 0.83 |
| Yes | 2968 (17.2) | 1786 (17.2) | 1182 (17.1) |  |
| No | 14316 (82.8) | 8585 (82.8) | 5731 (82.9) |  |
| Gastroparesis ever, N (%) |  |  |  | 0.95 |
| Yes | 184 (1.1) | 110 (1.1) | 74 (1.1) |  |
| No | 17100 (98.9) | 10261 (98.9) | 6839 (98.9) |  |
| Pancreatitis ever, N (%) |  |  |  | 0.20 |
| Yes | 435 (2.5) | 248 (2.4) | 187 (2.7) |  |
| No | 16849 (97.5) | 10123 (97.6) | 6726 (97.3) |  |
| Hypertension ever, N (%) |  |  |  | 0.16 |
| Yes | 11293 (65.3) | 6733 (64.9) | 4560 (66.0) |  |
| No | 5991 (34.7) | 3638 (35.1) | 2353 (34.0) |  |
| COPD or asthma ever, N (%) |  |  |  | 0.049 |
| Yes | 2420 (14.0) | 1496 (14.4) | 924 (13.4) |  |
| No | 14864 (86.0) | 8875 (85.6) | 5989 (86.6) |  |
| Cardiac dysrhythmias ever, N (%) |  |  |  | 0.32 |
| Yes | 2724 (15.8) | 1658 (16.0) | 1066 (15.4) |  |
| No | 14560 (84.2) | 8713 (84.0) | 5847 (84.6) |  |
| Malignant neoplasm ever, N (%) |  |  |  | 0.92 |
| Yes | 1277 (7.4) | 768 (7.4) | 509 (7.4) |  |
| No | 16007 (92.6) | 9603 (92.6) | 6404 (92.6) |  |
| Anemia ever, N (%) |  |  |  | 0.35 |
| Yes | 3587 (20.8) | 2128 (20.5) | 1459 (21.1) |  |
| No | 13697 (79.2) | 8243 (79.5) | 5454 (78.9) |  |
| Drug abuse, N (%) |  |  |  | 0.69 |
| Never | 14131 (81.8) | 8499 (81.9) | 5632 (81.5) |  |
| History | 2495 (14.4) | 1485 (14.3) | 1010 (14.6) |  |
| Current | 658 (3.8) | 387 (3.7) | 271 (3.9) |  |
| Current infection,^c^ N (%) |  |  |  | 0.5 |
| Yes | 3466 (20.1) | 2097 (20.2) | 1369 (19.8) |  |
| No | 13818 (79.9) | 8274 (79.8) | 5544 (80.2) |  |
| Current complication of device, graft, or implant, N (%) | |  |  | 0.25 |
| Yes | 414 (2.4) | 237 (2.3) | 177 (2.6) |  |
| No | 16870 (97.6) | 10134 (97.7) | 6736 (97.4) |  |
| Current fluid or electrolyte disorder, N (%) |  |  |  | 0.25 |
| Yes | 2849 (16.5) | 1737 (16.7) | 1112 (16.1) |  |
| No | 14435 (83.5) | 8634 (83.3) | 5801 (83.9) |  |

ACE-I, angiotensin converting enzyme inhibitor; ARB, angiotensin receptor blocker; COPD, chronic obstructive pulmonary disease; DKA, diabetic ketoacidosis; HHS, hyperglycemic hyperosmolar syndrome; IQR, interquartile range; ^a^Retinopathy, neuropathy, nephropathy; ^b^Coronary artery disease, heart failure, stroke, peripheral vascular disease; ^c^Pneumonia, urinary tract infection, septicemia, skin or subcutaneous infection;
