# Supplementary material for: Common sampling and modeling approaches to analyzing readmission risk that ignore clustering produce misleading results
Source: BMC Med Res Methodol. 2020 Nov 25;20:281. doi: 10.1186/s12874-020-01162-0 (PMC7687737; doi:10.1186/s12874-020-01162-0)
Supplement: Supplementary file 2 — Additional file 2: Supplementary Table 2. Characteristics of hospitalized patients with diabetes for all discharges by training and validation samples. [file 12874_2020_1162_MOESM2_ESM.docx]

**Supplementary Table 2 - Characteristics of hospitalized patients with diabetes for all discharges by training and validation samples**

| Variable | Total  N=44203 | Training  N=26402 | Validation  N=17801 | P value |
| --- | --- | --- | --- | --- |
| Age, N (%) |  |  |  | 0.45 |
| <50 years | 8455 (19.1) | 5152 (19.5) | 3303 (18.6) |  |
| 50-59 years | 10216 (23.1) | 5997 (22.7) | 4219 (23.7) |  |
| 60-69 years | 11466 (25.9) | 6945 (26.3) | 4521 (25.4) |  |
| 70+ years | 14066 (31.8) | 8308 (31.5) | 5758 (32.3) |  |
| Gender, N (%) |  |  |  | 0.8 |
| Female | 22286 (50.4) | 13275 (50.3) | 9011 (50.6) |  |
| Male | 21917 (49.6) | 13127 (49.7) | 8790 (49.4) |  |
| Marital status, N (%) |  |  |  | 0.29 |
| Married | 13780 (31.2) | 8064 (30.5) | 5716 (32.1) |  |
| Single | 29526 (66.8) | 17784 (67.4) | 11742 (66.0) |  |
| Other or unknown | 897 (2.0) | 554 (2.1) | 343 (1.9) |  |
| Race/ethcnicity, N (%) |  |  |  | 0.15 |
| Black | 20126 (45.5) | 12187 (46.2) | 7939 (44.6) |  |
| Hispanic | 6856 (15.5) | 4198 (15.9) | 2658 (14.9) |  |
| White | 14504 (32.8) | 8472 (32.1) | 6032 (33.9) |  |
| Other or unknown | 2717 (6.1) | 1545 (5.9) | 1172 (6.6) |  |
| English speaking, N (%) |  |  |  | 0.5 |
| Yes | 35858 (81.1) | 21487 (81.4) | 14371 (80.7) |  |
| No | 8345 (18.9) | 4915 (18.6) | 3430 (19.3) |  |
| Insurance status, N (%) |  |  |  | 0.37 |
| Medicaid | 9747 (22.1) | 5667 (21.5) | 4080 (22.9) |  |
| Medicare | 22118 (50.0) | 13300 (50.4) | 8818 (49.5) |  |
| None | 2297 (5.2) | 1414 (5.4) | 883 (5.0) |  |
| Private | 10041 (22.7) | 6021 (22.8) | 4020 (22.6) |  |
| Home zip code, N (%) |  |  |  | 0.73 |
| ≥5 miles from hospital | 13738 (31.1) | 8168 (30.9) | 5570 (31.3) |  |
| <5 miles from hospital | 30465 (68.9) | 18234 (69.1) | 12231 (68.7) |  |
| Education level, N (%) |  |  |  | 0.42 |
| Less than high school | 6058 (13.7) | 3518 (13.3) | 2540 (14.3) |  |
| Any high school | 24243 (54.8) | 14672 (55.6) | 9571 (53.8) |  |
| Some college | 2976 (6.7) | 1828 (6.9) | 1148 (6.4) |  |
| College graduate | 6724 (15.2) | 3920 (14.8) | 2804 (15.8) |  |
| Unknown | 4202 (9.5) | 2464 (9.3) | 1738 (9.8) |  |
| Employment, N (%) |  |  |  | 0.7 |
| Disabled | 9505 (21.5) | 5822 (22.1) | 3683 (20.7) |  |
| Employed | 4317 (9.8) | 2571 (9.7) | 1746 (9.8) |  |
| Retired | 16985 (38.4) | 9995 (37.9) | 6990 (39.3) |  |
| Unemployed | 12046 (27.3) | 7227 (27.4) | 4819 (27.1) |  |
| Other or unknown | 1350 (3.1) | 787 (3.0) | 563 (3.2) |  |
| Pre-admission sulfonylurea use, N (%) |  |  |  | 0.032 |
| Yes | 6697 (15.2) | 3839 (14.5) | 2858 (16.1) |  |
| No | 37506 (84.8) | 22563 (85.5) | 14943 (83.9) |  |
| Pre-admission metformin use, N (%) |  |  |  | 0.83 |
| Yes | 12400 (28.1) | 7387 (28.0) | 5013 (28.2) |  |
| No | 31803 (71.9) | 19015 (72.0) | 12788 (71.8) |  |
| Pre-admission thiazolidinedione use, N (%) |  |  |  | 0.5 |
| Yes | 3008 (6.8) | 1765 (6.7) | 1243 (7.0) |  |
| No | 41195 (93.2) | 24637 (93.3) | 16558 (93.0) |  |
| Pre-admission insulin use, N (%) |  |  |  | 0.28 |
| Yes | 16555 (37.5) | 10024 (38.0) | 6531 (36.7) |  |
| No | 27648 (62.5) | 16378 (62.0) | 11270 (63.3) |  |
| Pre-admission glucocorticoid use, N (%) |  |  |  | 0.41 |
| Yes | 4320 (9.8) | 2641 (10.0) | 1679 (9.4) |  |
| No | 39883 (90.2) | 23761 (90.0) | 16122 (90.6) |  |
| Most extreme blood glucose level, N (%) |  |  |  | 0.15 |
| 40-69 or 181-300 mg/dL | 19234 (43.5) | 11582 (43.9) | 7652 (43.0) |  |
| 70-180 mg/dL | 16128 (36.5) | 9479 (35.9) | 6649 (37.4) |  |
| <40 or >300 mg/dL | 8841 (20.0) | 5341 (20.2) | 3500 (19.7) |  |
| Diabetes inpatient consultation, N (%) |  |  |  | 0.86 |
| Yes | 5697 (12.9) | 3411 (12.9) | 2286 (12.8) |  |
| No | 38506 (87.1) | 22991 (87.1) | 15515 (87.2) |  |
| Current or prior DKA or HHS, N (%) |  |  |  | 0.48 |
| Yes | 3220 (7.3) | 1989 (7.5) | 1231 (6.9) |  |
| No | 40983 (92.7) | 24413 (92.5) | 16570 (93.1) |  |
| Microvascular complications,^a^ N (%) |  |  |  | 0.84 |
| 0 | 30854 (69.8) | 18488 (70.0) | 12366 (69.5) |  |
| 1 | 8227 (18.6) | 4873 (18.5) | 3354 (18.8) |  |
| 2 | 3294 (7.5) | 1917 (7.3) | 1377 (7.7) |  |
| 3 | 1828 (4.1) | 1124 (4.3) | 704 (4.0) |  |
| Macrovascular complications,^b^ N (%) |  |  |  | 0.47 |
| 0 | 19301 (43.7) | 11561 (43.8) | 7740 (43.5) |  |
| 1 | 12716 (28.8) | 7488 (28.4) | 5228 (29.4) |  |
| 2 | 8840 (20.0) | 5281 (20.0) | 3559 (20.0) |  |
| 3 | 2647 (6.0) | 1595 (6.0) | 1052 (5.9) |  |
| 4 | 699 (1.6) | 477 (1.8) | 222 (1.2) |  |
| Pre-admission BP meds, N (%) |  |  |  | 0.998 |
| None | 12274 (27.8) | 7325 (27.7) | 4949 (27.8) |  |
| ACE-i or ARB | 21352 (48.3) | 12757 (48.3) | 8595 (48.3) |  |
| Non-ACE or ARB | 10577 (23.9) | 6320 (23.9) | 4257 (23.9) |  |
| Pre-admission statin use, N (%) |  |  |  | 0.65 |
| Yes | 21153 (47.9) | 12582 (47.7) | 8571 (48.1) |  |
| No | 23050 (52.1) | 13820 (52.3) | 9230 (51.9) |  |
| White blood cell count, N (%) |  |  |  | 0.82 |
| Low <4 K/μL | 2019 (4.6) | 1226 (4.6) | 793 (4.5) |  |
| Normal 4-11 K/μL | 33932 (76.8) | 20232 (76.6) | 13700 (77.0) |  |
| High >11 K/μL | 8252 (18.7) | 4944 (18.7) | 3308 (18.6) |  |
| Serum hematocrit (%), mean (SD) | 33.7 (5.29) | 33.7 (5.25) | 33.7 (5.34) | 0.80 |
| Serum albumin, N (%) |  |  |  | 0.52 |
| 4+ g/dL | 15234 (34.5) | 9024 (34.2) | 6210 (34.9) |  |
| <4 g/dL | 23982 (54.3) | 14368 (54.4) | 9614 (54.0) |  |
| Unknown | 4987 (11.3) | 3010 (11.4) | 1977 (11.1) |  |
| Serum sodium, N (%) |  |  |  | 0.029 |
| Low <135 mmol/L | 4743 (10.7) | 2730 (10.3) | 2013 (11.3) |  |
| Normal 135-145 mmol/L | 39026 (88.3) | 23431 (88.7) | 15595 (87.6) |  |
| High >145 mmol/L | 434 (1.0) | 241 (0.9) | 193 (1.1) |  |
| Serum potassium, N (%) |  |  |  | 0.09 |
| Low <3.1 mmol/L | 540 (1.2) | 302 (1.1) | 238 (1.3) |  |
| Normal 3.1-5.3 mmol/L | 40345 (91.3) | 24043 (91.1) | 16302 (91.6) |  |
| High >5.3 mmol/L | 3318 (7.5) | 2057 (7.8) | 1261 (7.1) |  |
| Serum creatinine (mg/dL), median (IQR) | 0.98 (0.7, 1.4) | 0.98 (0.7-1.4) | 0.98 (0.7, 1.4) | 0.81 |
| Body mass index, N (%) |  |  |  | 0.91 |
| <18.5 kg/m^2^ | 1038 (2.3) | 616 (2.3) | 422 (2.4) |  |
| 18.5 – 24.9 kg/m^2^ | 7412 (16.8) | 4405 (16.7) | 3007 (16.9) |  |
| 25.0 – 29.9 kg/m^2^ | 12377 (28.0) | 7452 (28.2) | 4925 (27.7) |  |
| ≥30.0 kg/m^2^ | 23376 (52.9) | 13929 (52.8) | 9447 (53.1) |  |
| Discharged 90 days prior to index admission, N (%) | |  |  | 0.65 |
| Yes | 14168 (32.1) | 8507 (32.2) | 5661 (31.8) |  |
| No | 30035 (67.9) | 17895 (67.8) | 12140 (68.2) |  |
| Discharge 1 year prior to index admission, N (%) |  |  |  | 0.78 |
| Home | 15798 (35.7) | 9379 (35.5) | 6419 (36.1) |  |
| Home with nursing care | 5792 (13.1) | 3430 (13.0) | 2362 (13.3) |  |
| Sub-acute facility | 5320 (12.0) | 3238 (12.3) | 2082 (11.7) |  |
| Against medical advice | 666 (1.5) | 384 (1.5) | 282 (1.6) |  |
| No discharge recorded | 16627 (37.6) | 9971 (37.8) | 6656 (37.4) |  |
| Urgent or emergent admission,^c^ N (%) |  |  |  | 0.94 |
| Yes | 38145 (86.3) | 22780 (86.3) | 15365 (86.3) |  |
| No | 6058 (13.7) | 3622 (13.7) | 2436 (13.7) |  |
| Intensive care admission, N (%) |  |  |  | 0.43 |
| Yes | 7078 (16.0) | 4265 (16.2) | 2813 (15.8) |  |
| No | 37125 (84.0) | 22137 (83.8) | 14988 (84.2) |  |
| Blood transfusion given, N (%) |  |  |  | 0.92 |
| Yes | 6052 (13.7) | 3610 (13.7) | 2442 (13.7) |  |
| No | 38151 (86.3) | 22792 (86.3) | 15359 (86.3) |  |
| Parenteral or enteral nutrition, N (%) |  |  |  | 0.37 |
| Yes | 1592 (3.6) | 927 (3.5) | 665 (3.7) |  |
| No | 42611 (96.4) | 25475 (96.5) | 17136 (96.3) |  |
| Depression or psychosis ever, N (%) |  |  |  | 0.77 |
| Yes | 13112 (29.7) | 7870 (29.8) | 5242 (29.4) |  |
| No | 31091 (70.3) | 18532 (70.2) | 12559 (70.6) |  |
| Gastroparesis ever, N (%) |  |  |  | 0.48 |
| Yes | 2100 (4.8) | 1196 (4.5) | 904 (5.1) |  |
| No | 42103 (95.2) | 25206 (95.5) | 16897 (94.9) |  |
| Pancreatitis ever, N (%) |  |  |  | 0.87 |
| Yes | 2387 (5.4) | 1413 (5.4) | 974 (5.5) |  |
| No | 41816 (94.6) | 24989 (94.6) | 16827 (94.5) |  |
| Hypertension ever, N (%) |  |  |  | 0.17 |
| Yes | 32532 (73.6) | 19290 (73.1) | 13242 (74.4) |  |
| No | 11671 (26.4) | 7112 (26.9) | 4559 (25.6) |  |
| COPD or asthma ever, N (%) |  |  |  | 0.30 |
| Yes | 10433 (23.6) | 6365 (24.1) | 4068 (22.9) |  |
| No | 33770 (76.4) | 20037 (75.9) | 13733 (77.1) |  |
| Cardiac dysrhythmias ever, N (%) |  |  |  | 0.45 |
| Yes | 10546 (23.9) | 6382 (24.2) | 4164 (23.4) |  |
| No | 33657 (76.1) | 20020 (75.8) | 13637 (76.6) |  |
| Malignant neoplasm ever, N (%) |  |  |  | 0.44 |
| Yes | 4406 (10.0) | 2688 (10.2) | 1718 (9.7) |  |
| No | 39797 (90.0) | 23714 (89.8) | 16083 (90.3) |  |
| Anemia ever, N (%) |  |  |  | 0.95 |
| Yes | 18264 (41.3) | 10916 (41.3) | 7348 (41.3) |  |
| No | 25939 (58.7) | 15486 (58.7) | 10453 (58.7) |  |
| Drug abuse, N (%) |  |  |  | 0.91 |
| Never | 35424 (80.1) | 21120 (80.0) | 14304 (80.4) |  |
| History | 6942 (15.7) | 4177 (15.8) | 2765 (15.5) |  |
| Current | 1837 (4.2) | 1105 (4.2) | 732 (4.1) |  |
| Current infection,^c^ N (%) | |  |  | 0.62 |
| Yes | 9975 (22.6) | 5989 (22.7) | 3986 (22.4) |  |
| No | 34228 (77.4) | 20413 (77.3) | 13815 (77.6) |  |
| Current complication of device, graft, or implant, N (%) | |  |  | 0.29 |
| Yes | 1820 (4.1) | 1052 (4.0) | 768 (4.3) |  |
| No | 42383 (95.9) | 25350 (96.0) | 17033 (95.7) |  |
| Current fluid or electrolyte disorder, N (%) |  |  |  | 0.61 |
| Yes | 8913 (20.2) | 5351 (20.3) | 3562 (20.0) |  |
| No | 35290 (79.8) | 21051 (79.7) | 14239 (80.0) |  |

^a^Retinopathy, neuropathy, nephropathy; ^b^Coronary artery disease, heart failure, stroke, peripheral vascular disease; ^c^Pneumonia, urinary tract infection, septicemia, skin or subcutaneous infection; COPD, chronic obstructive pulmonary disease
