# Supplementary material for: Common sampling and modeling approaches to analyzing readmission risk that ignore clustering produce misleading results
Source: BMC Med Res Methodol. 2020 Nov 25;20:281. doi: 10.1186/s12874-020-01162-0 (PMC7687737; doi:10.1186/s12874-020-01162-0)
Supplement: Supplementary file 3 — Additional file 3: Supplementary Table 3. Performance of Logistic Regression Using First Discharges (n = 6913), All Discharges (n = 17,801), All Discharges With GEE, and All Discharges With CWGEE to Predict All-Cause 30-Day Readmission Among Adults With Diabetes, Boston, Massachusetts, 2004–2012. [file 12874_2020_1162_MOESM3_ESM.docx]

**Supplementary Table 3. Performance of Logistic Regression Using First Discharges^a^ (n=6913), All Discharges^b^ (n=17801), All Discharges With GEE, and All Discharges With CWGEE to Predict All-Cause 30-Day Readmission Among Adults With Diabetes, Boston, Massachusetts, 2004-2012.**

| Performance Measure  (95% CI) | First Discharges | All Discharges | All Discharges with GEE | All Discharges with CWGEE |
| --- | --- | --- | --- | --- |
| Readmission Rate, % | 10.2  (9.5, 10.9) | 20.3  (19.8, 20.9) | | |
| ROCAUC | 0.821  (0.806, 0.835) | 0.811  (0.803, 0.818) | 0.803  (0.796, 0.811) | 0.791  (0.783, 0.798) |
| Coefficient of Discrimination, % | 15.0  (14.1, 15.9) | 21.0  (20.4, 21.6) | 18.4  (17.9, 19.0) | 15.8  (15.3, 16.3) |
| Correlation Measure | 0.37  (0.35, 0.39) | 0.45  (0.44, 0.46) | 0.48  (0.43, 0.45) | 0.42  (0.40, 0.43) |
| Brier Score | 0.080  (0.075, 0.084) | 0.129  (0.126, 0.132) | 0.132  (0.128, 0.135) | 0.135  (0.132, 0.139) |
| Scaled Brier Score | 0.132  (0.082, 0.182) | 0.197  (0.182, 0.220) | 0.183  (0.166, 0.207) | 0.159  (0.142, 0.184) |

GEE, generalized estimating equations; CI, confidence interval; CWGEE, cluster-weighted generalized estimating equations; ROCAUC, receiver operating characteristic area under the curve

^a^The first discharges approach analyzed the first index discharge per patient; ^b^The all discharges approach analyzed all index discharges per patient
