# Supplementary material for: Common sampling and modeling approaches to analyzing readmission risk that ignore clustering produce misleading results
Source: BMC Med Res Methodol. 2020 Nov 25;20:281. doi: 10.1186/s12874-020-01162-0 (PMC7687737; doi:10.1186/s12874-020-01162-0)
Supplement: Supplementary file 4 — Additional file 4: Supplementary Table 4. Change Over Sample Size of Logistic Regression Model Performance Measures Using First Discharges (n = 6913), All Discharges (n = 17,801), All Discharges With GEE, and All Discharges With CWGEE to Predict All-Cause 30-Day Readmission in Validation Sample of Adults With Diabetes, Boston, Massachusetts, 2004–2012. [file 12874_2020_1162_MOESM4_ESM.docx]

**Supplementary Table 4. Change Over Sample Size of Logistic Regression Model Performance Measures Using First Discharges^a^ (n=6913), All Discharges^b^ (n=17801), All Discharges With GEE, and All Discharges With WGEE to Predict All-Cause 30-Day Readmission in Validation Sample of Adults With Diabetes, Boston, Massachusetts, 2004-2012.**

| Change in performance measure/1000 patients  (95% CI)  P-value | First Discharges | All Discharges | All Discharges with GEE | All Discharges with CWGEE |
| --- | --- | --- | --- | --- |
| Coefficients of Discrimination, % | 0.107  (-0.045, 0.258)  P = 0.16 | 0.113  (-0.038, 0.264)  P = 0.14 | 0.285  (0.134, 0.437)  P = 0.0004 | 0.265  (0.113, 0.416)  P = 0.0009 |
| ROCAUC | 0.001  (-.0002,0.002)  P = 0.083 | 0.001  (-.0003,0.002)  P = 0.12 | 0.0025  (0.001, 0.0038)  P = 0.0002 | 0.003  (0.0019, 0.004)  P < 0.0001 |
| Correlation Measures | 0.0018  (-.00001,0.004)  P = 0.051 | 0.0017  (-.0001,0.004)  P = 0.059 | 0.0032  (0.001, 0.005)  P = 0.0006 | 0.0031  (0.001, 0.0049)  P = 0.0011 |
| Brier Score | 0.00002  (-.0004, 0.0005)  P = 0.08 | -0.0004  (-.0008, 0.00001)  P = 0.057 | -0.00077  (-.0012, -.0003)  P = 0.0007 | -0.00075  (-.0012, -.0003)  P = 0.0009 |
| Scaled Brier Score | -0.0002  (-.0032, .0028)  P = 0.9 | 0.0026  (-.0004, 0.0056)  P = 0.093 | 0.0048  (.0018, .0078)  P = 0.0024 | 0.0047  (.0016, .0077)  P = 0.0031 |

GEE, generalized estimating equations; CI, confidence interval; CWGEE, cluster-weighted generalized estimating equations; ROCAUC, receiver operating characteristic area under the curve

^a^The first discharges approach analyzed the first index discharge per patient; ^b^The all discharges approach analyzed all index discharges per patient
