# Supplementary material for: Common sampling and modeling approaches to analyzing readmission risk that ignore clustering produce misleading results
Source: BMC Med Res Methodol. 2020 Nov 25;20:281. doi: 10.1186/s12874-020-01162-0 (PMC7687737; doi:10.1186/s12874-020-01162-0)
Supplement: Supplementary file 5 — Additional file 5: Supplementary Table 5. Distribution of the Number of Discharges Per Patient (N = 17,801) in Sample of Adults With Diabetes, Boston, Massachusetts, 2004–2012. [file 12874_2020_1162_MOESM5_ESM.docx]

**Supplementary Table 5. Distribution of the Number of Discharges Per Patient (N=17801) in Sample of Adults With Diabetes, Boston, Massachusetts, 2004-2012.**

| **Number of discharges/patient** | **Patients, n** | **%** |
| --- | --- | --- |
|  |  |  |
| **1** | 9780 | 56.6 |
| **2** | 2936 | 17.0 |
| **3** | 1428 | 8.3 |
| **4** | 892 | 5.2 |
| **5** | 575 | 3.3 |
| **6** | 361 | 2.1 |
| **7** | 286 | 1.7 |
| **8** | 221 | 1.3 |
| **9** | 145 | 0.8 |
| **10** | 113 | 0.7 |
| **>10** | 547 | 3.2 |
